# Analysis of *Anoxybacillus* Genomes from the Aspects of Lifestyle Adaptations, Prophage Diversity, and Carbohydrate Metabolism

**DOI:** 10.1371/journal.pone.0090549

**Published:** 2014-03-06

**Authors:** Kian Mau Goh, Han Ming Gan, Kok-Gan Chan, Giek Far Chan, Saleha Shahar, Chun Shiong Chong, Ummirul Mukminin Kahar, Kian Piaw Chai

**Affiliations:** 1 Faculty of Biosciences and Medical Engineering, Universiti Teknologi Malaysia, Skudai, Johor, Malaysia; 2 Monash School of Science, Monash University Sunway Campus, Petaling Jaya, Selangor, Malaysia; 3 Division of Genetics and Molecular Biology, Institute of Biological Sciences, Faculty of Science, University of Malaya, Kuala Lumpur, Malaysia; 4 School of Applied Science, Temasek Polytechnic, Singapore; Wayne State University, United States of America

## Abstract

Species of *Anoxybacillus* are widespread in geothermal springs, manure, and milk-processing plants. The genus is composed of 22 species and two subspecies, but the relationship between its lifestyle and genome is little understood. In this study, two high-quality draft genomes were generated from *Anoxybacillus* spp. SK3-4 and DT3-1, isolated from Malaysian hot springs. *De novo* assembly and annotation were performed, followed by comparative genome analysis with the complete genome of *Anoxybacillus flavithermus* WK1 and two additional draft genomes, of *A. flavithermus* TNO-09.006 and *A. kamchatkensis* G10. The genomes of *Anoxybacillus* spp. are among the smaller of the family *Bacillaceae*. Despite having smaller genomes, their essential genes related to lifestyle adaptations at elevated temperature, extreme pH, and protection against ultraviolet are complete. Due to the presence of various competence proteins, *Anoxybacillus* spp. SK3-4 and DT3-1 are able to take up foreign DNA fragments, and some of these transferred genes are important for the survival of the cells. The analysis of intact putative prophage genomes shows that they are highly diversified. Based on the genome analysis using SEED, many of the annotated sequences are involved in carbohydrate metabolism. The presence of glycosyl hydrolases among the *Anoxybacillus* spp. was compared, and the potential applications of these unexplored enzymes are suggested here. This is the first study that compares *Anoxybacillus* genomes from the aspect of lifestyle adaptations, the capacity for horizontal gene transfer, and carbohydrate metabolism.

## Introduction

**Figure 1:**
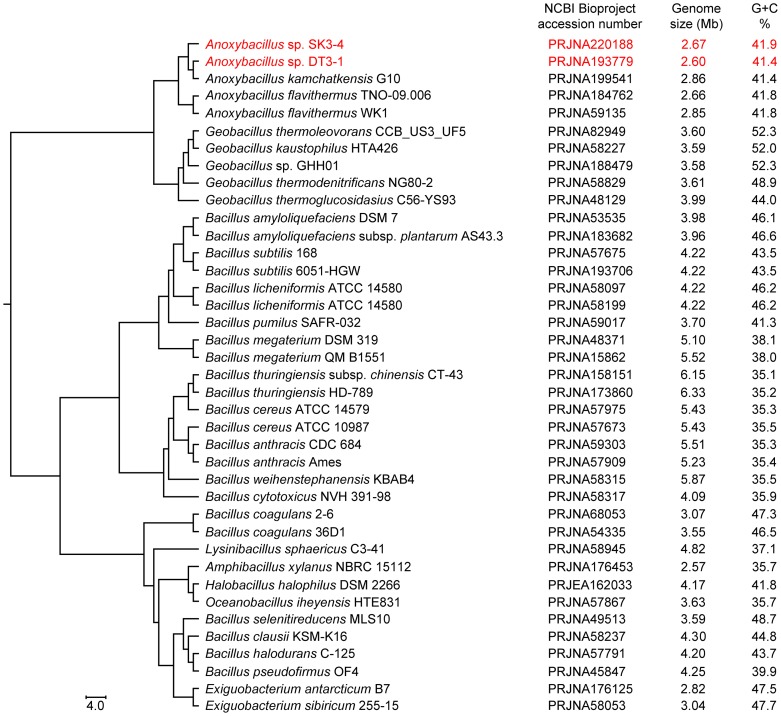
Phylogenetic tree of selected *Bacillaceae* based on concatenated sequences of 361 orthologs.

The *Bacillaceae* family remains an important microbial contributor to industrial biotechnology, mainly due to its abundance of useful proteins and enzymes [1]. All species within the family are Gram-positive and in the shape of a rod or coccus. More than 20,000 strains are classified as *Bacillaceae*, and the genomic knowledge about its genera is unequally distributed. Based on the National Center for Bioinformatics Information (NCBI) database, the accumulated genome sequencing projects registered for *Bacillus* and *Geobacillus* represent more than 99% of the total sequenced genomes for *Bacillaceae*. Among the *Bacillaceae* genomes, *Amphibacillus xylanus* NBRC 15112 has the smallest size of 2.57 Mb (**Figure 1**) and the lowest total numbers of genes and proteins (2,489 and 2,411, respectively). On average, *Bacillus* genomes are larger in size, with genomes of approximately 5.4 Mb, and with total open reading frames (ORFs) of more than 5,000 coding sequences (CDSs). The genome G+C% of *Bacillaceae* members does not correlate with their optimum cell growth temperatures (OGTs). For instance, the average G+C% for thermophilic *Anoxybacillus* (OGT 50−62°C) is 41.6, and this is lower than that of the G+C% of mesophilic *Halobacillus* (OGT 10−50°C, G+C% 44.3) and *Salimicrobium* (OGT 30−37°C, G+C% 46.3). *Geobacillus* appears to be the closest genus to *Anoxybacillus* based on the 16S rRNA phylogeny [1] and concatenated sequence similarity (**Figure 1**).

A number of *Anoxybacillus* spp. have been isolated from around the world since the first introduction of the genus in 2000 [2]. To date, a total of 22 species and two subspecies of *Anoxybacillus* are described [1],[3],[4],[5]. The cells of *Anoxybacillus* spp. are generally rod-shaped and straight or slightly curved, often present in pairs or short chains, and they form endospores. Interestingly, *Anoxybacillus* spp. can be either alkaliphilic or alkalitolerant, and most of them are able to grow well at neutral pH. *Anoxybacillus* spp. are moderately thermophilic (OGT 50−62°C), with a slightly lower OGT than *Geobacillus* spp. (55−65°C). The *Anoxybacillus* spp. are either aerobes or facultative anaerobes. Among the *Anoxybacillus* spp., the genome of *A. flavithermus* WK1 (PRJNA59135) remains the only completely sequenced genome [6]. Recently, the draft genome of *Anoxybacillus flavithermus* TNO-09.006 (PRJNA184762) was introduced [7]. Both strains were isolated from dairy-processing plants in New Zealand and The Netherlands, respectively. Other draft genomes available in the NCBI database are *A. flavithermus* AK1 (PRJNA197764) and *A. flavithermus* Kn10 (PRJNA201846), the latter having been announced recently [8]. The draft genome of another species, *Anoxybacillus kamchatkensis* G10 (PRJNA199541), isolated from an Indonesian hot spring, was announced in 2012 [9]. In the current work, the genomes of two Malaysian *Anoxybacillus* spp. SK3-4 and DT3-1, were sequenced by whole-genome shotgun sequencing using Illumina MiSeq (California, USA). The strains were isolated from the Sungai Klah (3°59'47.88"N, 101°23'35.17"E) and Dusun Tua hot springs (3°8'13.5''N, 101°50'5.3''E) respectively, which are located 100 miles apart [10].

This is the first study to compare the genomes of various *Anoxybacillus* spp. The adaptation strategies of *Anoxybacillus* spp. to survive at elevated temperatures and mildly alkaline environments, as well as its nitrogen metabolism, are described. Although *Anoxybacillus* has one of the smallest genomes in *Bacillaceae* [6], we suggest that horizontal gene transfer (HGT) might be one of the adaptation strategies employed by *Anoxybacillus*. The presence of prophages in *Anoxybacillus* spp. has not been examined, but integrated phages were identified in this work. This study also provides an in-depth analysis of the glycoside hydrolase (GH) and suggests some interesting potential applications of these carbohydrate-metabolizing enzymes.

## Materials And Methods

### Determination Of Total Organic Content (toc) Of Hot Spring Water

The total organic content (TOC) of the water of Sungai Klah (SK) hot spring was analyzed using the APHA5310 B high-temperature combustion method [11]. The sample was vaporized at 680°C in a heated chamber packed with oxidative catalyst. The generated CO_2_ was then analyzed using a Shimadzu TOC-VCSH analyzer (Kyoto, Japan). The analyzer measured two paths, one for total carbon (TC) and one for inorganic carbon, and the TOC was determined by calculating the difference between the two values.

### Bacterial Strains And Growth Conditions

*Anoxybacillus* sp. SK3-4 and DT3-1 were grown in modified *Thermus* medium (4.0 g/L of peptone, 4.0 g/L of tryptone, 4.0 g/L of yeast extract, 2.0 g/L of NaCl, and 1.0 g/L of MgSO_4_ **•**7H_2_O) [10], unless specified. The culture was incubated at 55°C with rotary shaking at 200 rpm. For the experiment on nitrogen utilization in the medium, the culture was centrifuged at 8000 x *g* for 15 min at 4°C, and the cells were washed with 0.5% (w/v) NaCl solution. The cells were transferred into 100 ml medium (2.0 g/L of NaCl and 1.0 g/L of MgSO_4_ **•**7H_2_O) supplemented with the following nitrogen sources (4.0 g/L for each medium): tryptone, casamino acids, NaNO_3_, or NaNO_2_, in a 500 ml conical flask. The cell growth was determined by measuring the optical density at 600 nm (OD_600_) hourly. All experiments were performed at least in duplicate.

### Dna Isolation, Sequencing, And Annotation

**Figure 2:**
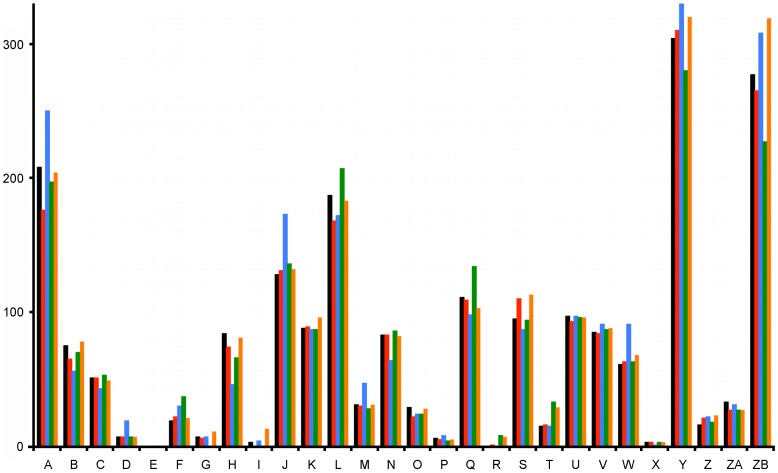
Subsystem feature counts according to the SEED classification. A: Cofactors, vitamins, prosthetic groups, pigments. B: Cell wall and capsule. C: Virulence, disease, and defense. D: Potassium metabolism. E: Photosynthesis. F: Miscellaneous. G: Phages, prophages, transposable elements, plasmids. H: Membrane transport. I: Iron acquisition and metabolism. J: RNA metabolism. K: Nucleosides and nucleotides. L: Protein metabolism. M: Cell division and cell cycle. N: Motility and chemotaxis. O: Regulation and cell signaling. P: Secondary metabolism. Q: DNA metabolism. R: Regulons. S: Fatty acids, lipids, and isoprenoids. T: Nitrogen metabolism. U: Dormancy and sporulation. V: Respiration. W: Stress response. X: Metabolism of aromatic compounds. Y: Amino acids and derivatives. Z: Sulfur metabolism. ZA: Phosphorus metabolism. ZB: Carbohydrates. Black: *Anoxybacillus* sp. SK3-4; red: *Anoxybacillus* sp. DT3-1; blue: *Anoxybacillus flavithermus* WK1; green: *Anoxybacillus flavithermus* TNO-09.006; orange: *Anoxybacillus kamchatkensis* G10.

**Figure 3:**
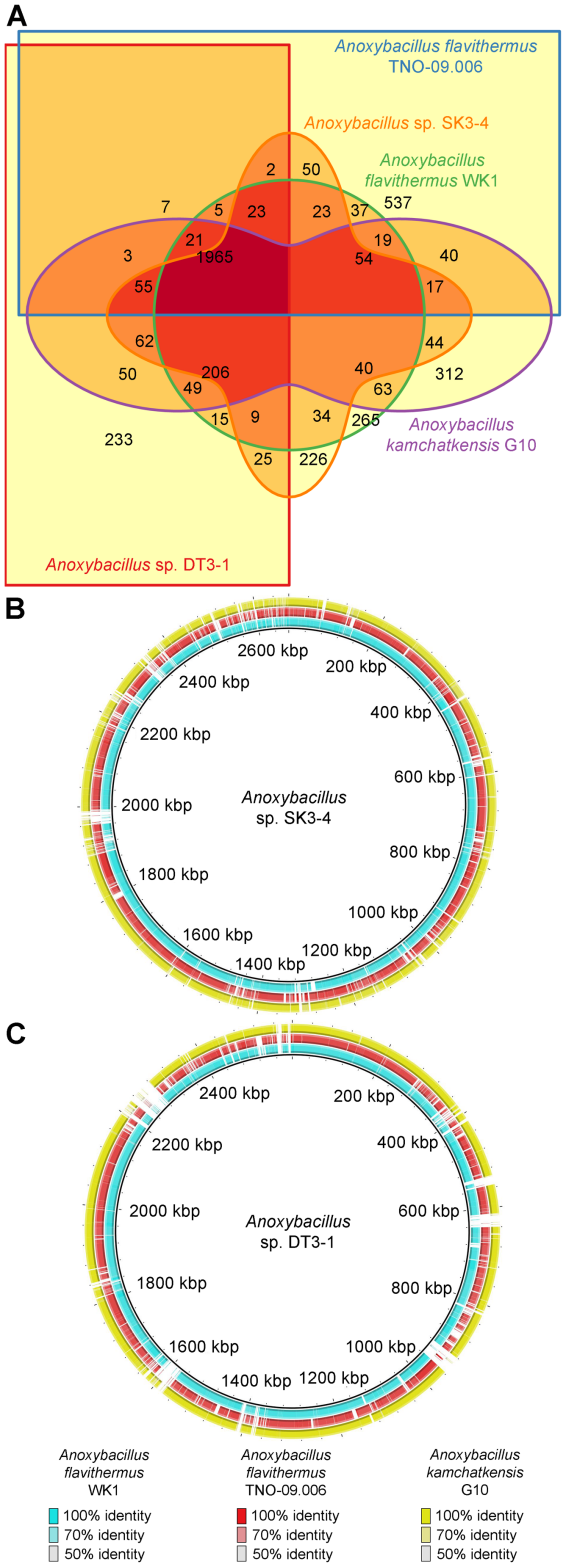
Genomes comparison of *Anoxybacillus* species. (**A**) Five-way Venn-diagram showing the number of shared and specific CDS among the *Anoxybacillus* spp. Orthologous groupings were based on 50% identify cutoff and overlap of at least 70% protein sequence length. (**B**) BRIG image with *Anoxybacillus* sp. SK3-4 genome sequence set as the central reference. (**C**) BRIG image with *Anoxybacillus* sp. DT3-1 genome sequence set as the central reference.

**Figure 4:**
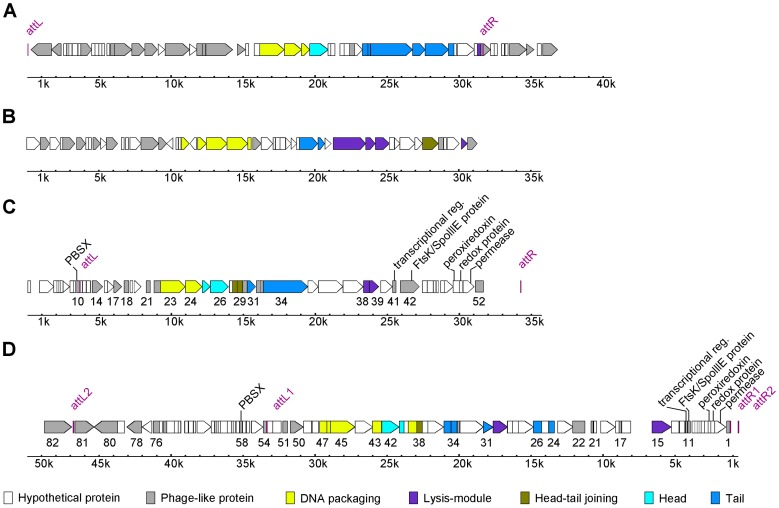
Sequence arrangements of prophages in *Anoxybacillus* genome as identified by PHAST. (**A**) ProphageWK of *Anoxybacillus flavithermus* WK1. (**B**) ProphageG10 of *Anoxybacillus kamchatkensis* G10. (**C**) ProphageSK of *Anoxybacillus* sp. SK3-4. (**D**) ProphageDT of *Anoxybacillus* sp. DT3-1. ProphageWK and prophageG10 may not be intact prophages, due to the lack of putative genes encoding morphological proteins. This is in contrast to intact prophageSK and prophageDT, which have more and ordered morphological genes. These morphological genes are arranged in clusters or modules, which is a hallmark of prophage sequences, and in an order typical of temperate tailed-phage genomes. Note that prophageDT is located on the complementary strand of *Anoxybacillus* sp. DT3-1. The prophage map was reversed for ease of reference. Both prophageSK and prophageDT also share six genes (shown in figure) that appear to be conserved in location and order. The details of ORFs information for prophageSK and prophageDT are provided in **Table S1** and **Table S2**.

**Figure 5:**
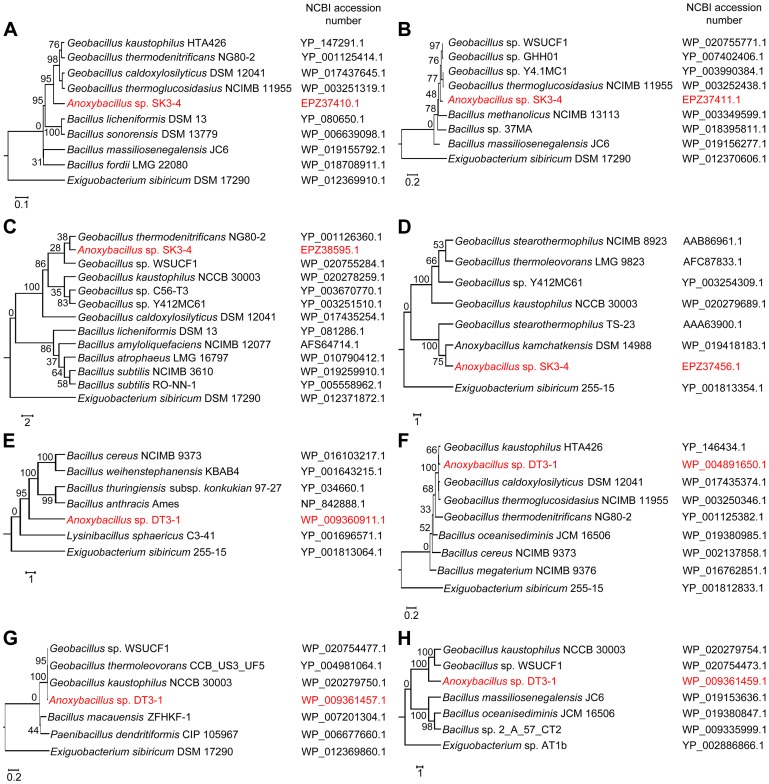
Protein dendrogram of sequences with the encoded genes as a result of horizontal gene transfer. (**A**) *Anoxybacillus* sp. SK3-4 proteinase, (**B**) *Anoxybacillus* sp. SK3-4 N-acetyltransferase, (**C**) *Anoxybacillus* sp. SK3-4 β-glucosidase, (**D**) *Anoxybacillus* sp. SK3-4 α-amylase, (**E**) *Anoxybacillus* sp. DT3-1 Na^+^/H^+^ antiporter NhaC, (**F**) *Anoxybacillus* sp. DT3-1 Mn^2+^/Fe^2+^/Zn^2+^ transporter, (**G**) *Anoxybacillus* sp. DT3-1 Mn^2+^/Fe^2+^ transporter, and (**H**) *Anoxybacillus* sp. DT3-1 drug transmembrane transport.

The genomic DNA for *Anoxybacillus* spp. SK3-4 and DT3-1 was obtained using the Wizard Genomic DNA Purification kit (Promega, Wisconsin, USA). Samples were prepared in accordance with the Illumina protocol, and whole-genome shotgun sequencing was done using Illumina MiSeq platform (California, USA). *De novo* assembly and annotations were performed using CLC Genomics Workbench 4.8 (CLCBio, Aarhus, Denmark) and Blast2GO [12] programs, respectively. This whole-genome shotgun sequencing project has been deposited at DDBJ/EMBL/GenBank under the accession numbers ANOC00000000 and ANMT00000000, respectively, for *Anoxybacillus* spp. SK3-4 and DT3-1. The versions described in this paper are versions ANOC01000000 and ANMT01000000. The Bioproject numbers for *Anoxybacillus* sp. DT3-1 are PRJNA193779 and PRJNA182115, and that for *Anoxybacillus* sp. SK3-4 are PRJNA220188 and PRJNA174378. To analyze the genomes of other *Anoxybacillus* spp., the complete sequences of *A. flavithermus* WK1 (PRJNA59135) and *A. flavithermus* TNO-09.006 (PRJNA184762) and the un-annotated contigs of *A. kamchatkensis* G10 (PRJNA199541) were obtained from NCBI. Other software used in this study included RNAmmer [13], tRNAscan-SE [14], InterProScan [15], Blast Ring Image Generator; BRIG [16], PanOCT [17], PHAge Search Tool; PHAST [18], Rapid Annotations using Subsystems Technology; RAST [19], SEED [20], and dbCAN carbohydrate-active enzymes; dbCAN CAZy [21]. For **Figure 1**, PanOCT was used to cluster the genes using a 50% identity cutoff and a 70% minimum aligned length. Using these parameters, a core genome for *Bacillaceae* strains across representatives from 361 orthologs was identified. These orthologs were aligned using ClustalW 2.0 [22] and eventually concatenated for phylogenomics analysis. The default settings for PanOCT were used, unless otherwise stated. The additional information in **Figure 1** (i.e., accession numbers, genome sizes, and G+C%) was obtained from NCBI’s BioProject database and was added manually to the figure. The SEED analysis (**Figure 2**) was done using the RAST database [19],[20]. The Venerable package in R was used to construct the five-way Venn diagram (**Figure 3A**) using a 50% identity cutoff. PHAST [18] was used to identify the putative prophages in *Anoxybacillus* genomes **(** **Figure 4** **).** To create the phylogenetic tree shown in **Figure 5**, unique sequences that were most likely due to horizontal gene transfer were searched against NCBI’s nucleotide database, and the most similar sequences were aligned with ClustalW 2.0. Subsequently, neighbor-joining trees were inferred using the MEGA5 software [23], and 1000 bootstrap replicates were performed.

## Results And Discussion

### General Features And Comparison Of Genomes Of Strains Sk3-4 And Dt3-1 With Other *Anoxybacillus* Genomes

In comparison to *Bacillaceae* members such as the *Bacillus* and *Geobacillus*, *Anoxybacillus* was somehow neglected when it was first identified as *Bacillus flavithermus* [1],[24]. At present, *A. flavithermus* WK1 is the only *Anoxybacillus* species with a completely sequenced genome [6], despite the completion of many other genomes from *Bacillus* and *Geobacillus* spp. Recently, several *Anoxybacillus* spp. were sequenced using the shotgun approach, and this motivated us to compare them. So far, no plasmid from these *Anoxybacillus* spp. has been examined, though members of *Bacillaceae* commonly have one or multiple plasmids. General features of the analyzed genomes are listed in **Table 1**. The five genomes fall into the range of 2.6 to 2.9 Mb, smaller than the closest genus, *Geobacillus* (**Figure 1**). The whole-genome, rRNA and tRNA G+C% across the *Anoxybacillus* spp. were more or less similar. The total number of predicted protein-coding genes in the *Anoxybacillus* spp. SK3-4 and DT3-1 draft genomes was 2,842 and 2,736, respectively. The draft genome of *A. kamchatkensis* G10 had approximately 100 more protein-coding genes than the completed genome of *A. flavithermus* WK1. The average protein length in *Anoxybacillus* spp. SK3-4 and DT3-1 was 284 amino acids, which was similar to *A. flavithermus* strains WK1 and TNO-09.006, and *A. kamchatkensis* G10.

**Table 1:** Genome features of the *Anoxybacillus* species used in this study.

	*Anoxybacillus* sp. SK3-4	*Anoxybacillus* sp. DT3-1	*Anoxybacillus flavithermus* WK1	*Anoxybacillus flavithermus* TNO-09.006	*Anoxybacillus kamchatkensis* G10
NCBI Bioproject accession number	PRJNA220188	PRJNA193779	PRJNA59135	PRJNA184762	PRJNA199541
Size (bp)	2,671,589	2,596,638	2,846,746	2,651,725	2,858,657
Contigs	148	52	1	68	65
N50 (bp)	75,006	153,724	2,846,746	65,120	130,036
G+C content (%)	41.89	41.46	41.78	41.82	41.35
G+C content of rRNA (%)	55.80	56.24	55.68	57.66	56.33
G+C content of tRNA (%)	59.43	60.03	59.47	59.33	59.40
Protein coding genes	2,842	2,736	2,863	2,595	2,953
Proteins with gene ontology	1,935	1,884	2,526	1,796	1,996
Total rRNA genes	3	5	24	10	6
Total tRNA genes	71	57	77	62	56
Reference	This study	This study	[6]	[7]	[9]

**Figure 2** shows the SEED analysis using the RAST database [19],[20]. A majority of the protein-encoded sequences functioned in the catabolic pathways of amino acids and their derivatives and carbohydrates. The total sequences of *A. flavithermus* WK1 involved in the functions of cofactors, vitamins, prosthetic group, pigments, potassium metabolism, RNA metabolism, cell division and cell cycle, and amino acids are higher than the other four genomes (**Figure 2**). It was initially thought that *Anoxybacillus* spp. from natural heated springs would exhibit more stress response sequences, but unexpectedly, milk processing effluent *A. flavithermus* WK1 had the most such genes. In comparison, the total genes for cell wall and capsule, membrane transport, motility and chemotaxis, and DNA metabolism in *A. flavithermus* WK1 were fewer than in the other four strains.

All five *Anoxybacillus* spp. shared the same morphology-related genes. The *Anoxybacillus* cells are rod-shaped, and each species exhibited an operon of three rod-shape-determining proteins (*Anoxybacillus* sp. SK3-4: C289_0313−0315, *Anoxybacillus* sp. DT3-1: F510_1310−1311) and a cell-shape-determining Mbl protein (C289_1660, F510_1488). The latter protein exhibited similarity of higher than 80% to *Bacillus* and *Geobacillus* rod-shape-determining protein MreB. Though these proteins have not been studied in regard to *Anoxybacillus* cell morphogenesis, the high level of similarity suggests a conserved role of the proteins in other closely related genera within *Bacillaceae* [25].

The genes that encode the flagellar assembly are complete and include various ring, motor protein, hook, and filament sequences. This suggests that the five *Anoxybacillus* spp. are motile, despite earlier experimental motility tests that suggested that *Anoxybacillus* spp. SK3-4 and DT3-1 were non-motile in certain growth media [10]. It is unclear at this moment whether *Anoxybacillus* spp. adopts polar or peritrichous flagellation because no electron photomicrographs for these five strains or any other *Anoxybacillus* are available. *A. flavithermus* WK1, *Anoxybacillus* spp. SK3-4 and DT3-1 are spore-forming bacteria. Sporulation in *Bacillus subtilis* involves over 500 genes [26],[27]. In *Anoxybacillus*, the total genes involved are fewer than 100, yet their number still falls within the expected range due to the size of the genome of *Anoxybacillus* [27]. The sporulation feature of *Anoxybacillus* is one of the proposed reasons why the bacteria are frequently found in milk powder samples [1].

A Venn diagram (**Figure 3A**) was used to compare the orthologous gene complements of the five *Anoxybacillus*. BRIG data were generated for strains SK3-4 and DT3-1 as the central sequence, while other genomes were set as concentric rings (**Figure 3B and 3C**). The analysis showed that both strains exhibited certain regions that were not present in other *Anoxybacillus* genomes (**Figure 3B and 3C**). A part of the unique regions was contributed by the prophage, while some other sequences were possibly a result of HGT.

### Prophage Sequences In *Anoxybacillus* Genomes

To date, there is no detailed report of prophage sequences in the *Anoxybacillus*, although the presence of a prophage is acknowledged, at least in *A. flavithermus* WK1 [6]. Analysis of the five *Anoxybacillus* genomes using the PHAST program, [18] identified at least one complete prophage sequence in all strains except for *A. flavithermus* TNO-09.006. Several incomplete prophages or prophage remnants were also present. For this article, only putative intact prophages suggested by PHAST will be shown. In general, all intact prophages in *Anoxybacillus* spp. SK3-4 and DT3-1, *A. flavithermus* WK1, and *A. kamchatkensis* G10 showed low conservation (**Figure 4**). The prophageWK1 and prophageG10 carried a limited number of prophage structural genes that were interspersed with hypothetical proteins. This could suggest severe rearrangement that left behind non-functional prophage remnants despite being identified as intact by PHAST. Therefore, these two prophages remnants will not be further addressed here.

Determining the relationship between two prophages is not easy due to the high recombination rate that prophages exhibit. Attempts to identify a relationship between prophages have been based on several conserved prophage proteins. Two of these are the larger subunit of terminase and tail tape measure protein [28]. Based on the low similarities of the two prophage proteins from *Anoxybacillus* spp. SK3-4 and DT3-1, we believe that these two prophages are not related to each other (**Tables 2** **, S1, and S2**). Analysis of phages isolated from both strains would confirm this hypothesis.

**Table 2:** Protein sequence comparison of prophage large terminase and phage tail-measure protein.

Large terminase protein similarity (%)													
	SK3-4	DT3-1	WK1	G10	A	B	C	D	E	F	G	H	I
SK3-4	100	27	22	27	55	55	56	22	22	29	28	28	26
DT3-1		100	23	99	22	22	23	25	25	64	62	94	63
WK1			100	24	20	20	20	91	91	23	24	22	22
G10				100	22	22	23	25	25	64	62	94	63

Phage tail-measure protein similarity (%)													
	SK3-4	DT3-1	WK1	G10	J	K	L	M	N	F	G	O	P
SK3-4	100	14	31	26	12	32	15	20	31	41	26	43	35
DT3-1		100	15	13	45	12	24	10	11	15	14	18	13
WK1			100	24	17	51	21	34	24	48	39	53	30
G10				100	12	17	43	14	37	35	49	36	22

A: *Bacillus cereus* VD154 (gi|446509997). B: *Bacillus cereus* AH1272 (gi|488006653). C: *Bacillus thuringiensis* (gi|384182753). D: *Bacillus virus* 1 (gi|155042934). E: *Geobacillus* phage GBSV1 (gi|115334627). F: *Geobacillus thermoglucosidasius* C56-YS93 (gi|336234248). G: *Geobacillus* sp. Y412MC52 (gi|261418101). H: *Geobacillus* sp. Y4.1MC1 (gi|312109743). I: *Paenibacillus mucilaginosus* K02 (gi|511629193). J: *Bacillus cereus* AH1271 (gi|488002245). K: *Bacillus cereus* VD156 (gi|446819371). L: *Bacillus sonorensis* L12 (gi|493686871). M: *Bacillus methanolicus* PB1 (gi|490573477). N: *Geobacillus kaustophilus* HTA426 (gi|56419074). O: *Geobacillus virus* E2 (gi|148747742). P: *Clostridium ljungdahlii* DSM 13528 (gi|300853566).

The intact prophage sequence from *Anoxybacillus* sp. SK3-4, prophageSK, was identified in contig 46 with a G+C% of 41.77, close to the 41.89 of the host genome. In general, prophages have a G+C content that is different from that of the host bacterial genome. However, similar G+C content between the two genetic materials is not unusual. An example is the plasmid-like prophage vB-BcesS-IEBH of *Bacillus cereus* [29]. ProphageSK was approximately 25.4 kb and constituted less than 1% of the *Anoxybacillus* sp. SK3-4 total genome size. It consisted of 52 ORFs, 22 of which were putatively annotated as phage-like or prophage proteins (**Table S1**). Ten ORFs were morphogenic or encoded structural proteins for phage morphogenesis and could be classified into four functional modules or clusters. The clusters show an order of arrangement that is typical of temperate tailed-phage genomes: lysogeny–DNA packaging–head-tail joining–lysis [28] (**Figure 4C**). The same order of arrangement was also reported for prophage phIS3501 from *Bacillus thuringiensis* [30]. Meanwhile, integrase, a component of the lysogeny module, and holin, a component of the lysis module, were, as usual, located near each of the attachment sites that flanked the prophage sequence. A gene encoding bacterial ATPase, FtsK/SpoIIIE, was present between the lysis module and the 3’-attachment site. Ftsk is known for its DNA binding and transporting through membrane pores. Thus, the protein is responsible for equal DNA segregation during cell division and bacterial sporulation. Because sequence-similar FtsK/SpoIIIE is commonly found in DNA viruses, it is believed that this ATPase could function in viral DNA packaging as well [31]. The same possibility may apply to prophageSK.

Three ORFs were putatively annotated as non-structural or regulatory proteins (**Table S1**): phage-like element PBSX protein, phage-associated antirepressor, and a prophage transcriptional regulator. The phage-associated antirepressor and phage-like element PBSX protein were located outside of the prophageSK 5'-attachment boundary, where approximately 6 kb of phage-like sequences lay. Antirepressors counter effect the inhibition of viral structural proteins expression caused by repressor proteins, thus drives prophage induction. It would not be surprising if this antirepressor was involved in maintaining prophageSK, as others have reported of antirepressor that is functional although located in a genetic element outside of a prophage sequence [32]. Meanwhile, the function of phage-like element PBSX protein in prophage maintenance or induction remains undefined. It is our opinion that these proteins and the phage-like sequences outside of prophageSK are in fact a remnant of another prophage. This speculation is based on the prophage sequence from *Anoxybacillus* sp. DT3-1, which is described below.

On top of the phage-like or prophage proteins, three bacterial genes were found between the lysis module and the 3’-attachment site (**Figure 4C**). These were putatively annotated as peroxiredoxin family protein, a redox protein, and a permease. It is common for prophage sequences to be punctuated with bacterial genes, as prophages contain regions prone to chromosomal recombination [33]. Various classes of bacterial genes are commonly found in prophage sequences, one of them being antioxidation genes such as that encoding the peroxiredoxin/redox proteins. Because these genes are commonly found in bacterial scaffolds based on the neighboring sequences, as was the case for prophageSK, the three genes might have been laterally transferred from different bacteria [34].

Meanwhile, analysis of the genome of *Anoxybacillus* sp. DT3-1 identified a 48.9 kb intact prophage, prophageDT, in contig 20. Interestingly, closer examination showed the presence of two sets of attachment sites, suggesting that prophageDT could be made up of two prophages that are nested into one (**Figure 4D**). The first prophage, prophageDT-a, was shorter and resided within the longer, second prophage, prophageDT-b. Integration within another prophage is possible due to the presence of recombination areas in the sequences. Often, the second integration leads to recombination of two prophages or disruption of one of them [35]. In the case of prophageDT, the second integration by prophageDT-a seems to have caused disruption to prophageDT-b, leaving only a partial 5’-arm intact (**Table S2**). With this in mind, it is reasonable to believe the phage-like sequence outside of the 5’-attachment boundary of prophageSK is the remnant of another prophage. This is evidenced by the presence of a similar phage-associated antirepressor and phage-like element PBSX protein in the same 5′-end region of both prophages.

Intact prophageDT-a exhibited a total size of 32.6 kb. Together, prophageDT-a and prophageDT-b made up 1.25% of the *Anoxybacillus* sp. DT3-1 genome. Their G+C content of 40.52% was slightly lower than the 41.46% of host genome. ProphageDT-a contained morphogenic genes that were similar to prophageSK’s and arranged in the same order: lysogeny–DNA packaging–head-tail joining–lysis (**Figure 4D**). Compared to prophageSK, prophageDT-a had some gene redundancy that may have resulted from rearrangement or other unknown reasons. In these places, the head-tail joining cluster was disrupted by a second lysis module consisting of a hydrolase and a peptidoglycan-binding LysM protein. A second integrase was also present further from the expected lysogeny module. An Ftsk/SpoIIIE protein similar to that in prophageSK was present at the 3′-arm of the prophage sequence. Impressively, the whole arm between the lysis module and 3′-attachment site was identical to the 3′-arm of prophageSK.

### Even With Small Genomes, Gene Transfer Occurs In *Anoxybacillus*

Several sequences in the *Anoxybacillus* spp. SK3-4 and DT3-1 were unique among all *Anoxybacillus* spp. The stretch of sequence in locus C289_2549−2555 of *Anoxybacillus* sp. SK3-4 encoded various proteins that showed high similarity to their *Geobacillus* counterparts. C289_2549 was annotated as an intracellular proteinase (sharing 79.3% identity to *Geobacillus caldoxylosilyticus* DSM 12041) (**Figure 5A**), while C289_2550 was possibly acetyltransferase of the GNAT family (89.2% identity to *Geobacillus thermoglucosidasius* NCIMB 11955) (**Figure 5B**). C289_2552 had 93% identity to a nitrilase/cyanide hydratase and apolipoprotein n-acyltransferase of *G. thermoglucosidasius*. The subsequent genes in this stretch encoded an HxlR family transcriptional regulator, a glucose-6-phosphate 1-dehydrogenase, and a 6-phosphogluconate dehydrogenase, which were 92.5%, 87.6%, and 94% identical to *G. thermoglucosidasius*, respectively. In addition, the short contig 26 of *Anoxybacillus* sp. SK3-4 consisted of three ORFs (C289_1346−1348) whose closest blast hits were *Geobacillus thermodenitrificans* NG80-2 β-glucosidase (94.7%) (**Figure 5C**), a hypothetical protein (98.7%), and a transposase (94.9%). Additionally, *Anoxybacillus* sp. SK3-4 possessed an α-amylase gene (C289_2507) (**Figure 5D**), suspected to have resulted from lateral gene transfer from *Geobacillus* spp. (see subsequent section for details). We ruled out the possibility of DNA contaminations during the Illumina sequencing, as these sequences were also identified when we performed genome sequencing of *Anoxybacillus* sp. SK3-4 using an Ion-Torrent 318-chip (data not published). Moreover, a *Bacillus* like Na^+^/H^+^ antiporter, NhaC (F510_0421), was present in *Anoxybacillus* sp. DT3-1 (**Figure 5E**). A stretch of putative transporter genes (F510_0993, 0994, and 0996) was identified, and these sequences might have originated from *Geobacillus* spp. (**Figure 5F−H**). Their hypothetical function is possibly related to cation efflux, metal ion transport, and drug transmembrane transport, respectively.

In addition to its own inherited genome, these foreign sequences are likely to be active and play roles in the metabolism and survival of *Anoxybacillus*. So far, from the current analysis, most of the possible HGT genes had apparent *Geobacillus* origins. On average, the proteins of *Anoxybacillus* and *Geobacillus* were approximately 90% similar, indicating a gene similarity high enough to allow integration into the genome via homologous recombination. Both *Anoxybacillus* spp. SK3-4 and DT3-1 had the operon of the late competence proteins ComEC, ComEB and ComEA, and ComER (C289_1719−1722, F510_2326−2329), competence proteins ComGA−ComGG (C289_1814−1820, F510_2245−2239), and competence protein FC (C289_2397, F510_2421). The gene-knockout of similar competence proteins has shown that these proteins are involved in the gene uptake of *B. subtilis* [36], and their function is assumed to be similar in *Anoxybacillus*. Additionally, the natural genetic competence in *A. flavithermus* strains WK1 and TNO-09.006 could be controlled by the quorum-sensing ComP-ComA, which sense the signaling molecule ComX. The sequence similarity of ComX, ComP, and ComA between *A. flavithermus* strains WK1 and TNO-09.006 was 98%, 92%, and 85%, respectively. A similar operon is present in various types of *Geobacillus* spp. and *Bacillus* spp.

### Genome Stabilization

For *Anoxybacillus* spp. SK3-4 and DT3-1 to survive at their optimal growth temperature of 55°C [10], they must adopt certain survival strategies, which include the maintenance of structure and function of nucleic acids. We will focus on *Anoxybacillus* spp. SK3-4 and DT3-1 because the DNA-stabilizing approaches are well conserved in *A. flavithermus* WK1, *A. flavithermus* TNO-09.006, and *A. kamchatkensis* G10, unless specified. Preserving the secondary structure of DNA *in vivo* is usually related to the G+C% and the torsion constraints of the two DNA strands. The G+C% of *Anoxybacillus* spp. SK3-4 and DT3-1 was 41.89% and 41.46%, respectively, which was lower than expected. However, the G+C% of rRNA sequences of *Anoxybacillus* spp. SK3-4 and DT3-1 were 55.80% and 56.24%, respectively, which were higher than expected. Interestingly, the G+C% of tRNA sequences of *Anoxybacillus* spp. SK3-4 and DT3-1 were also higher than expected, 59.43% and 60.03%, respectively. Similar trends were observed for *A. flavithermus* WK1, *A. flavithermus* TNO-09.006, and *A. kamchatkensis* G10 (**Table 1**). As reviewed by Trivedi et al., the G+C% in rRNA and tRNA sequences are higher in thermophiles, though a lack of consistency in this high G+C% in protein-coding genes of thermophiles was noted [37]. Principle component analysis (PCA) of *Geobacillus kaustophilus* and other *Bacillus*-related species has revealed that high G+C% of rRNA is one possible thermophilic signature for *Geobacillus* [38]. High G+C% could result in protection of DNA from thermodegradation, though this alone could not provide thermal stability to DNA [37].

Another reason why *Anoxybacillus* is able to live in hot springs is due to the presence of reverse gyrase. *Anoxybacillus* spp. SK3-4 and DT3-1 were found to have a gene coding for primosome assembly protein (PriA; C289_0740, F510_0088). Reverse gyrase, a type 1A topoisomerase, is a common feature of hyperthermophilic *Archaea* and *Eubacteria* that is not present in mesophiles, and the enzyme maintains the genome stability at high temperature [37],[39]. Furthermore, positive supercoiling is able to stabilize the secondary structure of DNA. Future biochemical and functional confirmation via gene-knockout may provide greater insight into the role of this protein as a possible trait of *Anoxybacillus* in thermoadaptation. Whether reverse gyrase is a prerequisite for thermophiles such as *Anoxybacillus* or *Geobacillus* remains unknown. In fact, there is no accepted explanation why reverse gyrase is important to thermophiles [40]. We think that no single factor could be responsible for the thermophily of *Anoxybacillus*. Therefore, how *Anoxybacillus* copes with the hot spring environment should be addressed.

Polyamines are positively charged compounds that can bind to DNA and RNA and stabilize the latter [41]. *Anoxybacillus* spp. SK3-4 and DT3-1 possessed the biosynthesis pathway of linear polyamines, such as spermidine and spermine. Genome annotations revealed the presence of genes coding for spermidine synthases (C289_0840, F510_0514) and spermidine transport proteins (C289_0889, C289_0890, C289_0891, C289_0892; F510_563, F510_564, F510_565, F510_566). The enzymes were mapped to Kyoto Encyclopedia of Genes and Genomes, KEGG pathways (map 330), which indicated that spermidine and spermine contributed to the thermoadaptation traits of both strains. The genome of *Anoxybacillus* spp. SK3-4 and DT3-1 also revealed the presence of a gene coding agmatinase (C289_839, F510_513), which is involved in the production of agmatine. This corroborates the polyamine profiling by high performance liquid chromatography (HPLC) and gas chromatography (GC) that has revealed that spermidine and spermine are the major polyamines of *A. flavithermus*, *Anoxybacillus gonensis*, and *Anoxybacillus voinovskiensis* cultured at 50−65°C [42]. Agmatine is also detected in *A. flavithermus* and *A. voinovskiensis*. Additionally, putrescine and thermopentamine are found in *A. voinovskiensis* [42].

The presence of histone-like proteins in *Anoxybacillus* spp. SK3-4 and DT3-1 may also provide thermal stability to their genomes. Both strains have the gene that codes for DNA-binding protein HU-alpha (C289_1583, F510_2065), which is a 90-amino-acid histone-like protein. Coded by the *hupA* gene, the protein in *Anoxybacillus* spp. is hypothesized to be involved in wrapping DNA to stabilize it against denaturation and degradation under extreme environmental conditions. The DNA-binding protein HU from hyperthermophilic *Thermotoga maritime* bends DNA and constrains negative DNA supercoils in the presence of topoisomerase I. This unique architectural function may generate stable and compact aggregates that organize and protect genomic DNA [43].

### Dna Repair

*Anoxybacillus* spp. SK3-4 and DT3-1 exploits almost all known DNA repair mechanisms in maintaining the primary structure of DNA against heat, ultra-violet (UV) irradiation, and other stresses, such as deamination and depurination. These mechanisms in *A. flavithermus* WK1 can be accessed online through the BioCyc database [44]. To explain the DNA repair strategy used in *Anoxybacillus*, the emphasis will be placed on *Anoxybacillus* spp. SK3-4 and DT3-1. The enzymes involved in base excision repair (BER) include DNA glycosylases (formamidopyrimidine-DNA glycosylase C289_0211, F510_1414; uracil-DNA glycosylase C289_0859, F510_0533; A/G-specific adenine glycosylase C289_2446, F510_0363; and 3-methyladenine DNA glycosylase C289_0413, F510_0344), and these enzymes act on the damaged or altered bases by removing them. The remaining apurinic/apyrimidinic (AP) sites and deoxyribose phosphate residues are excised, respectively, by endonuclease (C289_1773, F510_2286) and enzymes with phosphodiesterase activity that are found in both strains. DNA polymerase then repairs the gap within the genome. For nucleotide excision repair (NER), the trimer sequences of UvrA-UvrB-UvrA (C289_0131−0132, F510_1941−1942) were found in *Anoxybacillus* spp. SK3-4 and DT3-1. The stretch of damaged DNA is cut by an UvrC (C289_0254, F510_1371) and is separated from the intact genome DNA molecule with a helicase (UvrD, C289_1503, F510_1978).

Both *Anoxybacillus* spp. SK3-4 and DT3-1 also exhibited genes related to the photo-reactivation (PR) DNA repair pathway. These included the proteins photoproduct (thymine dimer) lyase (C289_1832, F510_2228) and a deoxyribodipyrimidine photolyase (C289_2706, F510_0958). The PR system removes a DNA lesion that is caused by the long-term exposure to UV [45]. Genome analysis of *Anoxybacillus* sp. DT3-1 revealed the presence of genes coding for phytoene dehydrogenases (F510_1530, F510_1535−1536) and phytoene synthetase (F510_1534), which are enzymes involved in the biosynthesis of carotenoid. In general, carotenoids function in photoprotection. The presence of these genes revealed an interesting aspect of metabolic activities of *Anoxybacillus* sp. DT3-1 to adapt to the Malaysian equatorial climate that receives sunlight throughout the year. Additionally, the colonies of *Anoxybacillus* sp. DT3-1 are yellow in color [10], which could be due to the presence of carotenoid, as described in *A. flavithermus* WK1 [6]. Similarly, the genome of *A. flavithermus* WK1 also revealed the presence of carotenoid biosynthesis genes. On the other hand, *Anoxybacillus* sp. SK3-4 produced creamy-white colonies, indicating the absence of carotenoid biosynthesis genes.

Genes that encoded mismatch repair (MMR) genes were also found. Examples of the important MMR enzymes are DNA mismatch repair proteins MutS and MutL (C289_1132−1133, F510_2660−2661), DNA helicases (C289_1757, C289_1228, F510_0226, F510_1755), exonuclease VII (C289_1857, F510_2204), ssDNA-specific exonuclease (C289_0349, F510_1276), ssDNA DNA-binding protein (C289_0909, F510_0599), DNA polymerase III holoenzyme (C289_0194, C289_0649, C289_0926, C289_2575, C289_1717, C289_2686, F510_1083, F510_0179, F510_0616, F510_0200, F510_2324, F510_0296), and NAD-dependent DNA ligase LigA (C289_1758, F510_0225).

*Anoxybacillus* spp. also had an alkyl transfer (AT) DNA repair mechanism, as the gene that encoded O6-methylguanine-DNA methyltransferase was detected in *Anoxybacillus* spp. SK3-4 and DT3-1 (C289_2132, F510_1632). Lastly, *Anoxybacillus* spp. seemed to have the homologous recombination repair (HRR) pathway, as recombination proteins RecA (C289_1120, F510_2671) and RecR (C289_2577, F510_0198), Holliday junction ATP-dependent DNA helicases (RuvB, C289_0338, F510_1287), and RuvA (C289_0337, F510_1288) were present. The HRR system is important for the maintenance of chromosome integrity in response to double-strand breaks (DSBs). Due to the lack of necessary genes, all *Anoxybacillus* spp. as well as *Geobacillus* are unable to repair DSBs by direct ligation via the non-homologous end-joining (NHEJ) pathway. The *Anoxybacillus* DNA repair mechanism appears to be quite similar to that in mesophilic bacteria. The major machinery that is different between them is the enzyme involved. Thermophilic enzymes are tolerant to heat due to their favorable amino acid sequences, structure folding and inter- and intra-protein interactions. Stable and high-fidelity repair enzymes will definitely contribute to pushing the temperature limitation of *Anoxybacillus* above that of mesophiles.

### Adaptation To Temperature Shifts

Analysis of a pool of X-ray crystallization-determined protein structures suggested that proteins from thermophiles are richer in ionic interactions, hydrogen bonds, and certain amino acids, such as Arg and Tyr [46]. Because none of the *Anoxybacillus* proteins are structurally determined, we are unable to gain insight into the relationship between protein architecture and *Anoxybacillus* thermophily.

Although the optimum growth temperature for *Anoxybacillus* spp. SK3-4 and DT3-1 is 55°C, they were isolated from a site where the temperatures of the water fluctuate in the range of 50−80°C. We therefore believe that heat shock proteins play an important role in the folding, refolding or disaggregation of their proteins. Most of the *Anoxybacillus* chaperonin genes were arranged in a cluster. As an example, the heat shock protein Hsp70 (DnaK) works in the presence of ATP and Hsp40 (DnaJ, J-protein). Hsp70 and Hsp40 were located near each other (C289_1707−1709, F510_2313−2315). According to the EBI InterProScan analysis [15], DnaJ was a typical type I J-protein due to the presence of a J-domain at the N-terminus, flexible linker G/F region, zinc-finger domain, and chaperone domain at the C-terminus (data not shown). The dimeric GrpE, a co-chaperone for DnaK, was in the same gene cluster. A heat-inducible transcription repressor was located close to the gene cluster (C289_1710, F510_2316). Other proteins related to temperature adaptations were GroEL (a 60 kDa chaperonin) and its cochaperonin GroES (10 kDa chaperonin) (C289_1307−1308, F510_2630−2631), a few small Hsp20 molecular chaperones (C289_1527, C289_1738, F510_0245, F510_2005), Hsp33 (C289_2485, F510_0256), and ClpC (Hsp100; C289_1992), and its related Clp-protease (C289_0107, C289_1462, C289_2055, F510_1917, F510_2523, F510_2734).

*Anoxybacillus* spp. are able to grow slowly at 30°C [1]. For *Anoxybacillus* spp. to counteract the effect of a temperature decrease, two cold shock proteins (Csps) (C289_0502, C289_1164, F510_0693, F510_0789) are found in both *Anoxybacillus* spp. SK3-4 and DT3-1, as well as in other *Anoxybacillus* spp. genomes. The Csps are RNA chaperones that are important in destabilizing the secondary structures of RNAs and presumably facilitate transcription and translation [47]. Csps from *Escherichia coli* and *B. subtilis* are the most well-studied examples, and some work on the archaeal Csps has been reported as well [48]. Csps usually work in synergy with other proteins. As no transcriptome analysis is available, we cannot identify all the proteins involved in the cold-adaptation of *Anoxybacillus*.

### Adaptation To Alkaline Ph

Seven ORFs of Na^+^/H^+^ antiporter subunits A−G for *Anoxybacillus* spp. SK3-4 and DT3-1 were found as an operon (C289_2311−2317, F510_0629−0635) and were also present in the genomes of *A. kamchatkensis* G10, *A. flavithermus* strains WK1 and TNO-09.006. A possible transcriptional regulator and transcriptional antiterminator were found upstream and downstream of the operon, respectively. Unexpectedly, *Anoxybacillus* sp. DT3-1 possessed an antiporter NhaC (F510_0421) that was not present in the genomes of *Anoxybacillus* sp. SK3-4, *A. flavithermus* WK1, *A. flavithermus* TNO-09.006, and *A. kamchatkensis* G10. The protein sequence contained 472 residues and had a high similarity (90.5%) to the Na^+^/H^+^ antiporter NhaC of *B. cereus* BAG60-2. The up- and downstream-genes of this Na^+^/H^+^ antiporter NhaC shared an average similarity of 85% with the counterpart sequences from various *Bacillus* spp. and were absent in all analyzed *Anoxybacillus* spp. and *Geobacillus* spp.. *Anoxybacillus* sp. DT3-1 Na^+^/H^+^ antiporter NhaC might have originated from cohabitation of *Bacillus* genetic material via HGT. Despite the high temperature at the original geothermal site, a few naturally adapted *Bacillus* spp. were isolated when *Anoxybacillus* sp. DT3-1 was first obtained (data not published).

### Adaptation Of Nitrogen Metabolism

Based on the KEGG analysis, while the gene clusters of respiratory nitrate reductase (NarGHJI) and assimilatory nitrate reductase (NasBC) [49],[50],[51] were missing from the genomes of *Anoxybacillus* spp. SK3-4 and DT3-1, and *A. flavithermus* WK1, a complete set of NarGHJI was present in *A. kamchatkensis* G10 and *A. flavithermus* TNO-09.006 (**Table 3**). When compared with *Geobacillus* spp., out of ten *Geobacillus* genomes that were available in KEGG webpage, nine of them contained at least one complete NarGHJI or NasBC gene cluster, and five of them showed the presence of both clusters. At least one gene cluster (NarGHJI or NasBC) was found in the genomes of *Bacillus* spp., and four of these genomes had both NarGHJI and NasBC (**Table 3**). The proteobacterial type of nitrate reductase (NapAB) was not detected in the genome of any *Anoxybacillus* spp., *Geobacillus* spp. or *Bacillus* spp.

**Table 3:** Presence of gene clusters of NarGHJI and NasBC in *Anoxybacillus*, *Geobacillus*, and *Bacillus*.

Genus	Species	Gene cluster	
		NarGHJI	NasBC
*Anoxybacillus*	*Anoxybacillus* sp. SK3-4	−	−
	*Anoxybacillus* sp. DT3-1	−	−
	*Anoxybacillus flavithermus* WK-1	−	−
	*Anoxybacillus flavithermus* TNO	+	−
	*Anoxybacillus kamchatkensis* G10	+	−
*Geobacillus*	*Geobacillus kaustophilus*	−, NarI is present	+
	*Geobacillus thermodenitrificans*	−, NarI is present	+
	*Geobacillus thermoglucosidasius*	+	−
	*Geobacillus thermoleovorans*	+	+
	*Geobacillus* sp. WCH70	−, NarG and NarI is present	−
	*Geobacillus* sp. Y412MC61	+	+
	*Geobacillus* sp. Y412MC52	+	+
	*Geobacillus* sp. C56 T-3	+	+
	*Geobacillus* sp. Y4.1MC1	+	−
	*Geobacillus* sp. GHH01	+	+
*Bacillus*	*Bacillus subtilis* 168	+	+
	*Bacillus amyloliquefaciens* subsp. *plantarum* YAU B9601-Y2	+	+
	*Bacillus atrophaeus*	+	+
	*Bacillus halodurans*	−	+
	*Bacillus anthracis* Ames	+	−, NasB is present
	*Bacillus cereus* ATCC 14579	+	−, NasB is present
	*Bacillus clausii*	+	+
	*Bacillus pseudofirmus*	−	+
	*Bacillus megaterium* QM B1551	−	+
	*Bacillus cellulosilyticus*	−	+

+ present;

− absent.

Although a majority of the *Anoxybacillus* genomes lacked nitrate reductase gene clusters, all of them encoded a complete set of amino acid metabolism genes. In addition, proteases were detected in all five *Anoxybacillus* genomes. Therefore, it could be postulated that organic nitrogen compounds (amino acids, small peptides, and proteins) could be the preferred nitrogen source for the growth of *Anoxybacillus* spp. An attempt to grow *Anoxybacillus* spp. SK3-4 and DT3-1 in minimal medium supplemented with organic nitrogen compounds (casamino acids/tryptone) or inorganic nitrogen compounds (nitrate/nitrite) as the sole nitrogen source demonstrated that casamino acids/tryptone favored the growth of both strains. When casamino acids were used as the sole nitrogen source, the specific growth rate (µ) of *Anoxybacillus* spp. SK3-4 and DT3-1 was 0.48 h^−1^ and 0.83 h^−1^, respectively. In medium containing tryptone as the sole nitrogen source, the µ for *Anoxybacillus* spp. SK3-4 and DT3-1 was 0.60 h^−1^ and 1.19 h^−1^, respectively. Neither *Anoxybacillus* spp. SK3-4 and DT3-1 was able to use nitrate or nitrite as the nitrogen source for growth. Our hot spring water analyses revealed a total of 5.3 mg/L of organic nitrogen and only a trace amount of inorganic nitrogen compounds (less than 0.3 mg/L).

The *Anoxybacillus* genomes are among the smallest genomes in *Bacillaceae*, but the key metabolic nitrogen pathways are retained in the genomes of *Anoxybacillus*. *Anoxybacillus* spp. are mainly isolated from hot springs and milk-containing environments. Because these environments are rich in organic nitrogen compounds, the loss of nitrate reductase gene complexes that are responsible for inorganic nitrogen metabolism in the genomes of a majority of *Anoxybacillus* spp. is an effective and energy-saving strategy.

### Analysis Of Glycosyl Hydrolase (gh) Enzymes

As determined with the standard APHA5310 B method, the TOC in the water of SK hot spring was 9.0 mg/mL. Most of the carbon sources were not present in the form of simple reducing sugars that are ready for uptake by the cells. For the *Anoxybacillus* to obtain carbon, various glycosyl hydrolase (GH) enzymes are required. The SEED analysis showed that many proteins were involved in carbohydrate metabolism (**Figure 2**). This section intends to compare the GHs in *Anoxybacillus* spp. SK3-4 and DT3-1, and the other three known *Anoxybacillus* genomes. GH enzymes were identified using dbCAN CAZy [21].

The common GHs shared among the five analyzed genomes were those of families 1, 13, 23, 31, and 65 (**Table 4**), and the average protein sequence similarity was 93.5%. The amylopullulanase C289_2785 (a type II pullulanase, GH13, 2033 residues, MW 221.2 kDa) appeared to be the largest GH in *Anoxybacillus* sp. SK3-4 [52], *A. flavithermus* WK1, and *A. kamchatkensis* G10, and in fact the largest ORF in each of those genomes. In general, *Geobacillus* appeared to be the closest ortholog to most *Anoxybacillus* GHs. Surprisingly, five enzymes had sequences unique to *Anoxybacillus* spp. and not present in *Geobacillus* spp. These included GH1 β-glucosidase, GH31 α-glucosidase, GH32 sucrose-6-phosphate hydrolase, and GH65 sugar-hydrolase (C289_0782, F510_2484). They were closer (similarity of 53−94%) to their *Bacillus* and *Thermoanaerobacter* (57%) counterparts (**Table 4**).

**Table 4:** List of glycosyl hydrolases (GHs) in *Anoxybacillus* and their similarity to the other genera.

GH	Enzyme	Similarity within *Anoxybacillus* a					Sequence similarity to other genera (%)
		SK3-4	DT3-1	WK1	TNO-09.006	G10	
1	β-glucosidase	-	92.4	100	-	-	β-glucosidase from *Thermoanaerobacter pseudoethanolicus* ATCC 33223, 57%
	β-glucosidase	100	-	-	-	-	β-glucosidase from *Geobacillus kaustophilus* and *Geobacillus thermoleovorans*, 98%
2	Glycoside hydrolase	-	-	-	100	-	Glycoside hydrolase from *Geobacillus* sp. Y412MC52, 98%
13	Amylopullulanase	88.8	-	100	-	99.1	Amylopullulanase from *Geobacillus stearothermophilus* TS-23, 83%
	Pullulanase	90.5	89.5	100	95.6	89.4	Pullulanase from *Geobacillus thermoglucosidasius*, 59%
	α-amylase (cell-bound)	97.4	96.2	100	97.2	96.2	α-amylase from *Geobacillus* sp. WCH70, 73%
	α-amylase (extracellular)	100	-	-	-	95.0	α-amylase *Geobacillus stearothermophilus* TS-23, 94%
	Cyclomaltodextrinase	92.5	92.0	100	94.7	92.3	Maltogenic amylase from *Geobacillus stearothermophilus*, 74%
	Glycosidase	96.4	96.6	100	97.8	96.2	Oligo-1,6-glucosidase from *Geobacillus thermoglucosidasius*, 85%
	Oligo-1,4-1,6-alpha-glucosidase	96.0	96.0	100	98.1	96.0	Oligo-1,6-glucosidase from *Geobacillus thermoglucosidasius*, 71%
	Oligo-1,4-1,6-alpha-glucosidase	95.2	96.3	100	95.4	96.1	α-amylase catalytic region from *Geobacillus* sp. Y4.1MC1, 82%
	Trehalose-6-phosphate hydrolase	94.2	93.7	100	-	93.9	α-phosphotrehalase from *Geobacillus* sp. WCH70, 82%
	1,4-α-glucan branching enzyme	93.2	92.8	100	94.1	92.4	1,4-α glucan branching enzyme from *Geobacillus stearothermophilus*, 77%
23	Lytic murein transglycosylase	93.6	85.8	100	96.1	85.8	Lytic transglycosylase, *Geobacillus* sp. WCH70, 60%
31	α-glucosidase	91.0	89.2	100	88.1	89.2	α-glucosidase from *Bacillus thermoamyloliquefaciens*, 60%
32	Sucrase-6-phosphate hydrolase	91.3	91.8	100	91.0	91.3	β-fructosidase from *Bacillus megaterium*, 53%
	Sucrose-6-phosphate hydrolase	-	-	100	-	-	Sucrose-6-phosphate hydrolase from *Geobacillus* sp. Y4.1MC1, 64%
36	α-galactosidase	-	-	-	-	100	α-galactosidase from *Geobacillus stearothermophilus*, 81%
43	Glycosyl hydrolase	-	-	100	-	-	Putative exo-xylanase from *Geobacillus thermoleovorans*, 83%
51	α-L-arabinofuranosidase	-	-	100	-	-	α-L-arabinofuranosidase from *Geobacillus thermoleovorans*, 91%
52	β-xylosidase	-	-	-	-	100	β-xylosidase from *Geobacillus thermoglucosidasius*, 92%
65	Sugar hydrolase/phosphorylase	94.9	94.1	100	-	94.0	Kojibiose phosphorylase from *Bacillus thuringiensis*, 61%;
74	Glycosyl hydrolase BNR repeat-containing protein	-	100	-	97.0	-	Glycosyl hydrolase BNR repeat-containing protein from *Geobacillus thermoleovorans*, 89%

a The reference used in the protein sequence alignment is denoted as 100%.

Not all GHs recognized in reference genome *A. flavithermus* WK1 were found in the other four *Anoxybacillus* spp. For instance, the GH1 β-glucosidase (C289_1346) in strain SK3-4 had no homologue in other *Anoxybacillus* spp. but was 98% similar to the β-glucosidases in *G. kaustophilus* and *Geobacillus thermoleovorans*. Additionally, *Anoxybacillus* sp. SK3-4 (C289_2507) and *A. kamchatkensis* G10 exhibited an additional extracellular α-amylase that was not present in the other three genomes. This additional α-amylase was different from the cell-bound α-amylase (C289_0468) described earlier in *Anoxybacillus* sp. SK3-4 (or equivalent to *A. flavithermus* WK1 α-amylase in **Table 4**) [53]. The biochemical properties of the extracellular amylase (C289_2507) are unexplored at the time of writing; yet based on its amino acid sequence, the closest match is an amylase of *Geobacillus stearothermophilus* TS-23, with 94% similarity. On the other hand, *Anoxybacillus* sp. DT3-1 had an extra GH74 glycosyl hydrolase with a bacterial neuraminidase repeat (BNR) domain at the center of the sequence (F510_1955). The sequence was highly similar to the sequence in *A. flavithermus* TNO-09.006. The function of F510_1955 is unknown, but based on the InterProScan analysis [15], the N-terminus of the sequence had a signal peptide and a membrane lipoprotein lipid attachment site. F510_1955 also had a domain found to be an oligoxyloglucan-reducing end-specific cellobiohydrolase (OXG-RCBH).

**Figure 6:**
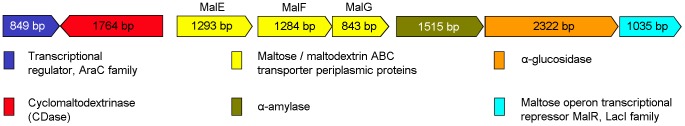
The gene cluster of various GHs, transporters, transcriptional regulators, and transcriptional repressors in *Anoxybacillus* sp. SK3-4. Identical clusters of genes are present in other *Anoxybacillus* species.

All of the GHs in the *Anoxybacillus* spp., in particular *Anoxybacillus* spp. SK3-4 and DT3-1, were not part of any gene cluster, except for the one shown in **Figure 6**. The gene cluster consisted of an intracellular α-glucosidase, a cell-anchoring α-amylase, three maltose/maltodextrin ABC transporter periplasmic proteins (MalE, MalF, and MalG; also known as permease), and an intracellular cyclomaltodextrinase (CDase). This gene cluster was sandwiched between a transcriptional regulator (AraC family) and the maltose operon transcriptional repressor MalR (LacI family) (C289_0463−0470, F510_1521−1528). The starch degradation process is most likely initiated by the action of the cell-anchoring α-amylase (C289_0468) and amylopullulanase (C289_2785), which hook to the cells by their transmembrane region and S-layer homology (SLH) domain, respectively, at their C-termini [52],[53]. The resulting oligosaccharides formed from the hydrolysis of starch are then transported into the cells by the maltose/maltodextrin ABC transporter periplasmic proteins and further degraded by the intracellular α-glucosidase and CDase. Although *A. flavithermus* CDase (*Af*Cda13) (77% identity to CDase from *Anoxybacillus* spp. SK3-4 and DT3-1) most actively acts on cyclodextrins, the enzyme can degrade starch into sugar [54],[55]. Without doubt, other, lone GHs (**Table 4**) help in the whole process of oligosaccharide degradation inside the cell.

We hope that knowing the types of GHs present in *Anoxybacillus* spp. will drive the discovery of new applications for thermostable enzymes in the starch industry. Many of the GHs listed in **Table 4** have not been biochemically characterized. For instance, the β-glucosidase in *Anoxybacillus* sp. DT3-1 (92.4% similar to *A. flavithermus* WK1) and a *Geobacillus*-similar β-glucosidase in *Anoxybacillus* sp. SK3-4 are yet to be explored. From the perspective of biofuel production, β-glucosidase (EC 3.2.1.21) is involved in the final step of converting cellobiose to glucose. It is an important enzyme, frequently known as a bottleneck enzyme in the hydrolysis of ligno-biomass [56]. In the food industry, β-glucosidases have various applications, such as to reduce the viscosity of gellan food, remove the bitterness of certain juices and unripe olives, and many other applications [57].

Another uncharacterized *Anoxybacillus* glucosidase is the α-glucosidase (synonym maltase, EC3.2.1.20, acts upon α-1, 4-glycosidic bonds). Although *Anoxybacillus salavatliensis* has shown an α-glucosidase activity [58], no α-glucosidase gene or its properties had been reported. In industry, α-glucosidase is mainly used to convert starchy substrates to glucose. In addition to α-glucosidase, oligo-1,4-1,6-alpha-glucosidase (synonym O16G, oligo-1,6-glucosidase, isomaltase, sucrase, EC 3.2.1.10) has not been examined for its biological function. According to BRENDA [59], O16G enzymes mainly attack the α-1,6-glucosidic linkages in isomaltose (two glucose units) or isomalto-oligosaccharides (2−6 glucose units), and certain reported O16Gs can hydrolyze the α-1, 6 bonds in starch, α-limit dextrins, and glycogen, resulting in the formation of maltose and linear or branched dextrins. This reactivity suggests that O16G from *Anoxybacillus* can serve as an alternative to pullulanase in assisting starch hydrolysis in industrial applications. We do not know why *Anoxybacillus*, for instance *Anoxybacillus* sp. SK3-4, requires numerous types of oligo-1,6-glucosidases, pullulanase type I, and amylopullulanase, in which all these enzymes can break identical α-1,6-glucosidic linkages. However, we think their presence is somehow related to the lifestyle of the cell. Apart from glucosidases, *Anoxybacillus* sp. DT3-1, *A. flavithermus* strains WK1 and TNO-09.006 each have a unique type of GHs (GH74, GH2, GH43, respectively) (**Table 4**). The efficiency of these glycosidases in degradation of biomass, such as cellulose and hemicellulose, has not been reported. Our group is currently analyzing some of these enzymes and hopes to determine the roles of each protein in the near future.

## Conclusion

*Anoxybacillus* is a mild thermophile. The closest genus to *Anoxybacillus* is *Geobacillus*, yet the genomes of the latter genus are larger and the cells grow at higher temperatures. Based on the genome annotation, the thermophily of *Anoxybacillus* is attributable to many features that stabilize proteins, DNA, and RNA. The presence of adaptive genes is sufficient for the cells to live in an alkaline environment with the presence of organic nitrogen and carbohydrates and to overcome the threats from UV radiation. In addition, for *Anoxybacillus* to survive under extreme conditions, we think that genetic exchange, especially uptake of genetic material via HGT, is important. Based on our genomic information, this process can take place via transduction or transformation, though at this moment, the possibility of HGT by conjugation remains undecided.

## Supporting Information

Table S1 **Database matches for prophageSK from** ***Anoxybacillus*** **sp. SK3-4**. 22 ORFs putatively annotated as phage-like or prophage proteins. Ten ORFs designated 23–26, 28, 29, 31, 34, 38, and 39 are involved in phage morphogenesis, while three ORFs designated 10, 14, and 41 are annotated as non-structural or regulatory proteins.
(DOC).

Table S2 **Database matches for prophageDT from** ***Anoxybacillus*** **sp. DT3-1**. ProphageDT is annotated on the complementary strand of contig 20 of *Anoxybacillus* sp. DT3-1. 32 ORFs putatively annotated as phage-like or prophage proteins. ORFs designated 1 to 53 form prophageDT-a, while ORFs 54 to 82 form the remnant of prophageDT-b.
(DOC).
