# Supplementary material for: Analysis of Anoxybacillus Genomes from the Aspects of Lifestyle Adaptations, Prophage Diversity, and Carbohydrate Metabolism
Source: PLoS One. 2014 Mar 6;9(3):e90549. doi: 10.1371/journal.pone.0090549 (PMC3948429; doi:10.1371/journal.pone.0090549)
Supplement: Table S1 — Database matches for prophageSK from Anoxybacillus sp. SK3-4. 22 ORFs putatively annotated as phage-like or prophage proteins. Ten ORFs designated 23–26, 28, 29, 31, 34, 38, and 39 are involved in phage morphogenesis, while three ORFs designated 10, 14, and 41 are annotated as non-structural or regulatory proteins. (DOC). [file pone.0090549.s001.doc]

**Table S1. Database matches for prophageSK from *Anoxybacillus* sp. SK3-4**. 22 ORFs putatively annotated as phage-like or prophage proteins. Ten ORFs designated 23–26, 28, 29, 31, 34, 38, and 39 are involved in phage morphogenesis, while three ORFs designated 10, 14, and 41 are annotated as non-structural or regulatory proteins

| **CDS No.** | **CDS Position** | **Representative similarity to protein in database (BLAST Hit)** | **E-Value** |
| --- | --- | --- | --- |
| 1 | 16−201 | PHAGE *Bacillus* virus 1: hypothetical protein BV1 gp53 | 8.00E-05 |
| 2 | 825−1817 | PHAGE *Staphylococcus* StauST398 3: hypothetical protein | 6.00E-37 |
| 3 | 1818−2027 | hypothetical protein GY4MC1 0600 (*Geobacillus* sp. Y4.1MC1) | 3.00E-18 |
| 4 | 2033−2194 | hypothetical protein GY4MC1 0601 (*Geobacillus* sp. Y4.1MC1) | 3.00E-12 |
| 5 | 2169−2291 | hypothetical protein GY4MC1 0602 (*Geobacillus* sp. Y4.1MC1) | 5.00E-07 |
| 6 | 2288−2455 | hypothetical protein GY4MC1 0603 (*Geobacillus* sp. Y4.1MC1) | 9.00E-19 |
| 7 | 2452−2934 | PHAGE *Geobacillus* virus E2: hypothetical protein GBVE2 gp049 | 6.00E-46 |
| 8 | 2936−3196 | PHAGE Phage OH2: hypothetical protein | 2.00E-19 |
| 9 | 3193−3330 | hypothetical protein (*Geobacillus thermoleovorans* CCB_US3_UF5) | 5.00E-09 |
| 10 | 3393−3602 | phage-like element PBSX protein (*Geobacillus* sp. Y4.1MC1) | 8.00E-28 |
|  | 3548−3561 | attL AACAGCGAAGGGAA |  |
| 11 | 3648−3818 | hypothetical protein GY4MC1 0605 (*Geobacillus* sp. Y4.1MC1) | 2.00E-16 |
| 12 | 3821−4051 | hypothetical protein Geoth 1117 (*Geobacillus thermoglucosidasius* C56-YS93) | 5.00E-06 |
| 13 | 4088−4339 | hypothetical | 0 |
| 14 | 4477−5208 | PHAGE *Bacillus* virus 1: phage associated-antirepressor | 1.00E-80 |
| 15 | 5340−5534 | PHAGE *Bacillus* phBC6A52: hypothetical protein BC2573 | 4.00E-07 |
| 16 | 5558−6001 | PHAGE *Bacillus* virus 1: hypothetical protein BV1 gp59 | 3.00E-41 |
| 17 | 5998−6540 | PHAGE *Bacillus* BtCS33: integrase | 4.00E-61 |
| 18 | 6691−6984 | PHAGE *Clostridium* phi3626: Gp47 protein | 6.00E-05 |
| 19 | 7128−7301 | hypothetical | 0 |
| 20 | 7403−7900 | hypothetical | 0 |
| 21 | 8243−8389 | hypothetical | 0 |
| 22 | 8794−9216 | PHAGE *Bacillus* virus 1: hypothetical protein BV1 gp17 | 5.00E-09 |
| 23 | 9223−10950 | PHAGE *Bacillus* phBC6A52: terminase large subunit | 0 |
| 24 | 10966−12126 | PHAGE *Bacillus* phBC6A52: portal protein | 2.00E-102 |
| 25 | 12123−12698 | PHAGE *Clostridium* phi3626: putative prohead protease | 3.00E-32 |
| 26 | 12695−13972 | PHAGE *Bacillus* phBC6A52: prohead protease | 1.00E-99 |
| 27 | 14005−14247 | PHAGE *Bacillus* phBC6A52: hypothetical protein BC2588 | 1.00E-15 |
| 28 | 14228−14554 | PHAGE *Geobacillus* virus E2: putative head-tail adaptor | 4.00E-24 |
| 29 | 14554−14943 | PHAGE *Clostridium* phiCD6356: putative head-tail joining protein | 3.00E-15 |
| 30 | 14955−15263 | PHAGE *Clostridium* phiCD6356: hypothetical protein | 4.00E-14 |
| 31 | 15275−15844 | PHAGE *Bacillus* BtCS33: major tail protein, phi13 family | 1.00E-33 |
| 32 | 15899−16186 | PHAGE *Bacillus* WBeta: conserved phage protein | 1.00E-12 |
| 33 | 16198−16365 | hypothetical protein GYMC61 0635 (*Geobacillus* sp. Y412MC61) | 9.00E-08 |
| 34 | 16379−19471 | PHAGE *Geobacillus* virus E2: putative tail tape measure protein | 0 |
| 35 | 19471−20202 | PHAGE *Bacillus* virus 1: hypothetical protein BV1 gp32 | 5.00E-77 |
| 36 | 20212−21900 | PHAGE *Bacillus* virus 1: hypothetical protein BV1 gp33 | 0 |
| 37 | 21911−23314 | PHAGE *Bacillus* virus 1: hypothetical protein BV1 gp34 | 0 |
| 38 | 23325−23738 | PHAGE *Bacillus* virus 1: Putative lysis protein | 4.00E-69 |
| 39 | 23735−24409 | PHAGE *Bacillus* virus 1: N-acetylmuramoyl-L-alanine amidase | 1.00E-100 |
| 40 | 24526−25335 | PHAGE *Geobacillus* virus E2: hypothetical protein GBVE2 gp025 | 1.00E-81 |
| 41 | complement (25363−25587) | PHAGE *Bacillus* virus 1: putative transcriptional regulator | 4.00E-34 |
| 42 | 25915−27234 | PHAGE *Bacillus* virus 1: FtsK/SpoIIIE family protein | 0 |
| 43 | 27434−27760 | PHAGE *Bacillus* virus 1: hypothetical protein BV1 gp40 | 2.00E-50 |
| 44 | 27776−28180 | PHAGE *Bacillus* virus 1: hypothetical protein BV1 gp41 | 3.00E-42 |
| 45 | 28192−28362 | PHAGE *Bacillus* virus 1: hypothetical protein BV1 gp42 | 2.00E-14 |
| 46 | 28424−28546 | hypothetical protein Aflv 2349 (*Anoxybacillus flavithermus* WK1) | 1.00E-13 |
| 47 | 28691−28990 | PHAGE Microm MpV1: hypothetical protein | 4.00E-06 |
| 48 | 29002−29577 | PHAGE Ostreo tauri virus 1: hypothetical protein H665 p205 | 9.00E-09 |
| 49 | 29574−29969 | peroxiredoxin family protein (*Anoxybacillus flavithermus* WK1) | 6.00E-66 |
| 50 | 29982−30209 | redox protein, regulator of disulfide bond formation (*Anoxybacillus flavithermus* WK1) | 1.00E-35 |
| 51 | 30258−31034 | permease (*Anoxybacillus flavithermus* WK1) | 6.00E-135 |
| 52 | 31117−31350 | PHAGE Mycoba Che12: gp59 | 3.00E-05 |
|  | 34254−34267 | attR AACAGCGAAGGGAA |  |
