# Supplementary material for: Analysis of Anoxybacillus Genomes from the Aspects of Lifestyle Adaptations, Prophage Diversity, and Carbohydrate Metabolism
Source: PLoS One. 2014 Mar 6;9(3):e90549. doi: 10.1371/journal.pone.0090549 (PMC3948429; doi:10.1371/journal.pone.0090549)
Supplement: Table S2 — Database matches for prophageDT from Anoxybacillus sp. DT3-1. ProphageDT is annotated on the complementary strand of contig 20 of Anoxybacillus sp. DT3-1. 32 ORFs putatively annotated as phage-like or prophage proteins. ORFs designated 1 to 53 form prophageDT-a, while ORFs 54 to 82 form the remnant of prophageDT-b. (DOC). [file pone.0090549.s002.doc]

**Table S2. Database matches for prophageDT from *Anoxybacillus* sp. DT3-1**. ProphageDT is annotated on the complementary strand of contig 20 of *Anoxybacillus* sp. DT3-1. 32 ORFs putatively annotated as phage-like or prophage proteins. ORFs designated 1 to 53 form prophageDT-a, while ORFs 54 to 82 form the remnant of prophageDT-b.

| **CDS No.** | **CDS Position** | **Representative similarity to protein in database (BLAST Hit)** | **E-Value** |
| --- | --- | --- | --- |
|  | 1−12 | attR2 AATCGAAAAAAA |  |
|  | 1198−1209 | attR1 TTATACCTTATA |  |
| 1 | complement (1233−1466) | PHAGE *Mycobacterium* Jasper: gp62 | 4.00E-06 |
| 2 | complement (1549−2325) | permease (*Anoxybacillus flavithermus* WK1) gi|212640166|ref|YP_002316686.1|; PP_00003 | 9.00E-134 |
| 3 | complement (2374−2601) | redox protein, regulator of disulfide bond formation (*Anoxybacillus flavithermus* WK1) | 1.00E-35 |
| 4 | complement (2613−3008) | peroxiredoxin family protein (*Anoxybacillus flavithermus* WK1) | 6.00E-66 |
| 5 | complement (3005−3580) | PHAGE Ostreo tauri virus 1: hypothetical protein H665 p205 | 9.00E-09 |
| 6 | complement (3592−3891) | PHAGE Microm MpV1: hypothetical protein | 2.00E-06 |
| 7 | complement (4036−4158) | hypothetical protein Aflv 2349 (*Anoxybacillus flavithermus* WK1) | 4.00E-13 |
| 8 | complement (4219−4389) | PHAGE *Bacillus* virus 1: hypothetical protein BV1 gp42 | 5.00E-15 |
| 9 | complement (4401−4805) | PHAGE *Bacillus* virus 1: hypothetical protein BV1 gp41 | 2.00E-41 |
| 10 | complement (4821−5279) | PHAGE *Bacillus* virus 1: hypothetical protein BV1 gp40 | 9.00E-74 |
| 11 | complement (5347−6666) | PHAGE *Bacillus* virus 1: FtsK/SpoIIIE family protein | 0 |
| 12 | 6995−7219 | PHAGE *Bacillus* virus 1: putative transcriptional regulator | 2.00E-31 |
| 13 | 7457−8158 | PHAGE Burkho phiE202: gp9, Cpp15 | 4.00E-21 |
| 14 | complement (8194−8769) | PHAGE *Listeria* B025: PlyB025 | 5.00E-46 |
| 15 | complement (8769−8969) | PHAGE *Clostridium* 39 O: holin | 2.00E-05 |
| 16 | complement (8969−9187) | PHAGE *Geobacillus* virus E2: hypothetical protein GBVE2 gp022 | 4.00E-13 |
| 17 | complement (9233−10228) | PHAGE *Thermus* P23 45: XerD-like integrase | 3.00E-23 |
| 18 | 10339−10500 | hypothetical | 0 |
| 19 | complement (10557−10688) | hypothetical | 0 |
| 20 | complement (10690−10887) | hypothetical | 0 |
| 21 | complement (10904−12178) | PHAGE *Pseudomonas* 201phi2 1: virion structural protein | 7.00E-07 |
| 22 | complement (12156−13223) | PHAGE *Vibrio* VHML: ORF37 | 1.00E-47 |
| 23 | complement (13487−13828) | hypothetical protein Sgly 3119 (*Syntrophobotulus glycolicus* DSM 8271) | 4.00E-22 |
| 24 | complement (13829−14323) | PHAGE Deep s D6E: tail fiber protein | 3.00E-18 |
| 25 | complement (14326−14898) | PHAGE Phage OH2: hypothetical protein | 1.00E-33 |
| 26 | complement (14895−15950) | PHAGE Phage OH2: baseplate J | 3.00E-70 |
| 27 | complement (15953−16366) | PHAGE Phage OH2: hypothetical protein | 1.00E-12 |
| 28 | complement (16366−16701) | hypothetical protein GY4MC1 0636 (*Geobacillus* sp. Y4.1MC1) | 2.00E-37 |
| 29 | complement (16706−17707) | PHAGE Phage OH2: hypothetical protein | 1.00E-24 |
| 30 | complement (17707−18360) | PHAGE *Clostridium* phiMMP02: peptidoglycan-binding LysM protein | 5.00E-30 |
| 31 | complement (18357−20027) | PHAGE *Clostridium* phiMMP04: tail tape measure | 3.00E-56 |
| 32 | complement (20039−20206) | hypothetical protein GY4MC1 0632 (*Geobacillus* sp. Y4.1MC1) | 1.00E-22 |
| 33 | complement (20227−20640) | PHAGE *Clostridium* phi CD119: XkdN protein | 9.00E-20 |
| 34 | complement (20686−21120) | PHAGE Phage OH2: core tail protein | 2.00E-06 |
| 35 | complement (21137−22204) | PHAGE *Clostridium* phiMMP04: tail sheath | 1.00E-61 |
| 36 | complement (22204−22623) | PHAGE *Clostridium* phiMMP04: hypothetical protein | 4.00E-13 |
| 37 | complement (22620−23015) | PHAGE Phage OH2: hypothetical protein | 3.00E-11 |
| 38 | complement (23015−23623) | head-tail joining family protein (*Geobacillus* sp. Y4.1MC1) | 1.00E-104 |
| 39 | complement (23620−23904) | PHAGE *Geobacillus* virus E2: putative DNA packaging protein | 2.00E-31 |
| 40 | complement (23936−24226) | PHAGE *Bacillus* virus 1: hypothetical protein BV1 gp22 | 2.00E-09 |
| 41 | complement (24245−25447) | PHAGE *Bacillus* WBeta: putative major capsid protein | 1.00E-111 |
| 42 | complement (25485−26087) | PHAGE *Bacillus* WBeta: putative *Caudovirales* phage prohead protease | 2.00E-85 |
| 43 | complement (26047−27333) | PHAGE *Bacillus* WBeta: putative phage portal protein | 7.00E-167 |
| 44 | complement (27350−27535) | hypothetical | 0 |
| 45 | complement (27540−29267) | PHAGE *Bacillus* BtCS33: phage terminase large subunit | 0 |
| 46 | complement (29264−29758) | PHAGE *Bacillus* BtCS33: phage terminase small subunit, P27 family | 2.00E-57 |
| 47 | complement (29867−30277) | PHAGE *Geobacillus* virus E2: Putative HNH endonuclease | 1.00E-46 |
| 48 | complement (30317−30877) | hypothetical protein Halha 2167 (*Halobacteroides halobius* DSM 5150) | 1.00E-21 |
| 49 | complement (30884−31801) | hypothetical protein EF2022 (*Enterococcus faecalis* V583) | 3.00E-22 |
| 50 | complement (32017−32310) | PHAGE *Clostridium* phi3626: Gp47 protein | 5.00E-06 |
| 51 | complement (32462−33004) | PHAGE *Bacillus* BtCS33: integrase | 4.00E-61 |
| 52 | complement (33001−33444) | PHAGE *Bacillus* virus 1: hypothetical protein BV1 gp59 | 4.00E-40 |
| 53 | complement (33468−33662) | PHAGE *Bacillus* phBC6A52: hypothetical protein BC2573 | 8.00E-07 |
|  | 33787−33798 | attL1 TTATACCTTATA |  |
| 54 | complement (33794−34525) | PHAGE *Bacillus* virus 1: phage associated-antirepressor | 1.00E-80 |
| 55 | complement (34655−34855) | hypothetical | 0 |
| 56 | complement (34898−35125) | hypothetical protein Geoth 1117 (*Geobacillus thermoglucosidasius* C56-YS93) | 8.00E-07 |
| 57 | complement (35131−35346) | hypothetical protein GY4MC1 0605 [*Geobacillus* sp. Y4.1MC1) | 2.00E-24 |
| 58 | complement (35347−35556) | phage-like element PBSX protein (*Geobacillus* sp. Y4.1MC1) | 8.00E-28 |
| 59 | complement (35628−35765) | hypothetical | 0 |
| 60 | complement (35774−35914) | hypothetical protein (*Geobacillus thermoleovorans* CCB_US3_UF5) | 1.00E-09 |
| 61 | complement (35911−36171) | PHAGE phage OH2: hypothetical protein | 6.00E-20 |
| 62 | complement (36173−36655) | PHAGE *Geobacillus* virus E2: hypothetical protein GBVE2 gp049 | 5.00E-46 |
| 63 | complement (36655−36819) | hypothetical protein GY4MC1 0603 (*Geobacillus* sp. Y4.1MC1) | 2.00E-19 |
| 64 | complement (36816−36938) | hypothetical protein GY4MC1 0602 (*Geobacillus* sp. Y4.1MC1) | 2.00E-07 |
| 65 | complement (36913−37074) | hypothetical protein GY4MC1 0601 (*Geobacillus* sp. Y4.1MC1) | 1.00E-12 |
| 66 | complement (37080−37280) | hypothetical protein GY4MC1 0600 (*Geobacillus* sp. Y4.1MC1) | 5.00E-20 |
| 67 | complement (37290−38282) | PHAGE *Staphylococcus aureus* StauST398 3: hypothetical protein | 3.00E-37 |
| 68 | complement (38390−38518) | hypothetical | 0 |
| 69 | complement (38549−38881) | PHAGE *Bacillus* virus 1: hypothetical protein BV1 gp53 | 1.00E-09 |
| 70 | complement (38886−39140) | hypothetical protein GY4MC1 0596 (*Geobacillus* sp. Y4.1MC1) | 9.00E-21 |
| 71 | complement (39415−39582) | hypothetical protein Aflv 0646 (*Anoxybacillus flavithermus* WK1) | 2.00E-05 |
| 72 | complement (39596−39820) | hypothetical | 0 |
| 73 | complement (39834−40355) | helix-turn-helix domain-containing protein (*Geobacillus* sp. Y4.1MC1) | 2.00E-86 |
| 74 | complement (40410−40586) | hypothetical protein GY4MC1 3261 (*Geobacillus* sp. Y4.1MC1) | 2.00E-21 |
| 75 | complement (40721−40918) | PHAGE *Listeria* A118: putative repressor protein | 6.00E-05 |
| 76 | 41070−41477 | PHAGE *Listeria* A118: putative repressor protein | 4.00E-14 |
| 77 | 41598−42296 | PHAGE *Staphylococcus* SA13: hypothetical protein | 2.00E-11 |
| 78 | 42313−43398 | PHAGE *Bacillus* SPBc2: putative DNase/RNase endonuclease | 1.00E-38 |
| 79 | 43493−43972 | hypothetical protein GY4MC1 0583 (*Geobacillus* sp. Y4.1MC1) | 7.00E-67 |
| 80 | 44044−45597 | PHAGE *Bacillus* SPBc2: site-specific recombinase | 5.00E-43 |
| 81 | complement (45594−46763) | PHAGE *Thermus* TMA: conserved bacterial protein | 4.00E-34 |
|  | 46878−46889 | attL2 AATCGAAAAAAA |  |
| 82 | complement (46984−48879) | PHAGE *Thermus* phiYS40: serine kinase | 2.00E-106 |
